# The Conference at Leicester

**Published:** 1919-07-26

**Authors:** 


### WOMAN'S WORLD.

### The Conference at Leicester.

ANYONE who supposed that the Parliamentary Suffrage when conceded would be the ultimate satisfaction of all women's aspirations, especially with the crowning gift of eligibility for membership, must soon feel disillusioned. The yearly conference of the National Council of Women, held this year at Leicester, marked as usual the progress of new developments. One session of very real interest was occupied with the position of women in law administration.

To begin with what may be considered the lower rungs of a great ladder reaching to the Woolsack, the work of women patrols, leading to that of women police, has been a success that surprises even those who hoped great things from the movement. Miss Peto, Director of the Bristol Training School, with which schools at Liverpool and Glasgow are now federated—gave an address which should have won recruits from among the younger listeners, coming as it did from a young woman and representing the work as far from negative and composed of *Don'ts*. The opportunities of preventing mischief by mere presence, and by drawing girls, and lads too, into organisations of all kinds, should appeal to V.A.D.s who are now looking round for new work. The training is almost absurdly cheap—£3 3s. for three to six months' instruction—while board and lodging can be arranged for at 25s. per week. There are also, thanks to the generosity of the Carnegie Trust, a number of free bursaries.

Immediate employment is practically certain for any satisfactory recruit at £100 to £150 a year for patrol leaders, who are employed in many places to manage a voluntary corps. Policewomen have at present a varying status, being sometimes sworn in and having varied powers, but their salary is not less than two to three guineas a week, plus uniform, for an eight-hour day, with one day off in seven or ten, and a fortnight's leave yearly on full pay. Candidates must be over twenty-five and not less than 5 ft. 4 in. in height, with good sight and hearing, and sound general health. They bind themselves to a minimum of two-years' service after training.

The woman probation officer has also proved her value. She is, for instance, often consulted by magistrates while cases are on remand. More women are also required as police-station matrons and so forth. The advisability of the woman magistrate was also discussed. This question is naturally interesting now when the mental and physical health of the delinquent child is a question that attracts much attention. Children's cases have been removed from the ordinary court with good results, and a suggestion is already heard that they should be remitted to the care of the Board of Education. "Benefit of Childhood" would then be our version of the old benefit of clergy. Nurses are likely to agree that justice administered to children needs to be of a maternal type. They know, too, the danger of self-consciousness; there is romance in being very naughty and having a great fuss made about it. The need to show life as the great adventure is a most pressing one in our education.

The prospect for a woman who aspires to the Bar—her aspiration being fitly voiced by Miss Helena Normanton—was not shown in a very encouraging light by Mejuffrouw Van Dorp, who has attained success in Holland. Prejudice against the woman pleader is still strong, she thinks, while the strain is great; but as an adviser in chambers she finds much usefulness. As in this country a judge is always a barrister of at least seven years' standing, the woman judge seems a remote figure.

A resolution was proposed by Miss Amy Hughes on behalf of Q.V.J.I.N. to the effect that the co-operation and help of district nurses should be asked in all public-health schemes, such as midwifery and infant welfare, the care of school-children and tuberculosis cases, on account of their intimate knowledge of the conditions of working-class life. Many of those present strongly felt that the district nurse's burden could ill bear any added strains, but that as adviser she should be priceless.

Are local nurses indeed sufficiently often included in social service committees? The question must have occurred to many during the Infant Welfare Conference at Kingsway Hall, when the grave question came up of the effect upon the pre-maturity death-rate, and upon feeble-mindedness and degeneracy of *unsuccessful* attempts at abortion. Important factors may be left out of consideration for want of the presence of those who know. This applies also to the statement made at this same Conference that private nurses know what a quantity of literature is posted both to father and mother when a birth appears in the newspapers. It seems likely that a serious contributing factor to the tale of infants lost to the nation has been overlooked hitherto.

